# Evaluating costs of heavy metal tolerance in a widely distributed, invasive butterfly

**DOI:** 10.1111/eva.13208

**Published:** 2021-04-03

**Authors:** Alexander M. Shephard, Amod M. Zambre, Emilie C. Snell‐Rood

**Affiliations:** ^1^ Department of Ecology, Evolution, and Behavior University of Minnesota – Twin Cities St. Paul MN USA

**Keywords:** anthropogenic change, butterfly, fitness cost, heavy metal pollution, invasive species, melanin, pest

## Abstract

Organismal tolerance to environmental pollution is thought to be constrained by fitness costs, where variants with higher survival in polluted environments have lower performance in nonpolluted environments. Yet, costs are not always detected in empirical studies. One hypothesis suggests that whether tolerance costs emerge depends on the degree of heterogeneity populations experience with respect to pollution exposure. For instance, in populations confined to local environments where pollution is persistent, selection may favour alleles that enhance pollution tolerance but reduce performance in nonpolluted environments (costs). However, in broadly distributed populations that undergo selection in both polluted and nonpolluted patches, costs should be eroded. Understanding tolerance costs in broadly distributed populations is relevant to management of invasive species, which are highly dispersive, wide ranging, and often colonize disturbed or polluted patches such as agricultural monocultures. Therefore, we conducted a case study quantifying costs of tolerance to zinc pollution (a common heavy metal pollutant) in wild cabbage white butterflies (*Pieris rapae*). This wide ranging, highly dispersive and invasive pest periodically encounters metal pollution by consuming plants in urban and agricultural settings. In contrast to expected costs of tolerance, we found that cabbage white families with greater zinc tolerance also produced more eggs and had higher reproductive effort under nonpolluted conditions. These results contribute to a more general hypothesis of why costs of pollution tolerance vary across studies: patchy selection with pollutants should erode costs and may favour genotypes that perform well under both polluted and nonpolluted conditions. This might partly explain why widely distributed invasive species are able to thrive in diverse, polluted and nonpolluted habitats.

## 1 INTRODUCTION

Human activities generate a range of stressful or toxic conditions that are often significantly different from conditions experienced by organisms in their evolutionary past. For instance, industry, agriculture and urbanization expose organisms to thousands of novel pollutants, ranging from heavy metals to pesticides to industrial compounds and ionizing radiation (Beresford & Copplestone, 2011; Stark & Banks, 2003; Wei & Yang, 2010). A common expectation among biologists is that adaptation to novel, polluted environments is accompanied by fitness costs of tolerance. Such costs are revealed when populations or genotypes that are relatively more tolerant to polluted environments (i.e. have a greater ability to survive pollutant exposure) have relatively lower fitness or reduced investment in components of fitness (e.g. fecundity, growth rate or body size) when exposed to a nonpolluted, ancestral environment (Hoffmann & Hercus, 2000; Hoffman & Parsons, 1991; Posthuma & Van Straalen, 1993; Sibly & Calow, 1989). With some notable exceptions (Lopes et al., 2008; McCart & Buckling, 2005), fitness costs have often been observed in populations that have undergone multiple generations of selection with exposure to relatively constant pollutant levels, typically in laboratory selection experiments (Abbas et al., 2012; Crow, 1957; Dutilleul et al., 2017; Jansen et al., 2011; Mireji et al., 2010; Shirley & Sibly, 2006; Ward & Robinson, 2005; Xie & Klerks, 2004). For instance, fitness costs of tolerance have been observed in populations of *Drosophila* (Shirley & Sibly, 2006) and killifish (Xie & Klerks, 2004) following multiple generations of laboratory selection under consistent heavy metal pollution and in arthropod species following laboratory selection for insecticide tolerance (Abbas et al., 2012; Cao & Han, 2006; Carrière et al., 2009; Shah et al., 2015; Wenes et al., 2006). In nature, while there is some evidence for selection against pollution‐tolerant genotypes in nonpolluted environments (Antonovics & Bradshaw, 1970; Levinton et al., 2003), fitness costs of pollution tolerance were shown to be weak or absent in some wild populations of plants (Harper et al., 1997; Rengel, 2000), aquatic invertebrates (Agra et al., 2011), soil invertebrates (Posthuma et al., 1992, 1993) and vertebrates (DiGiacopo & Hua, 2020). Yet, it remains unclear why fitness costs of tolerance vary in magnitude and are not always observed where expected.

To advance our understanding of why costs of pollution tolerance are not universally observed in populations, we need to consider the factors that may influence their variability. One hypothesis suggests that whether costs of tolerance emerge depends on the degree of heterogeneity populations experience with respect to pollution exposure (Hoffman & Parsons, 1991; Morgan et al., 2007). For instance, in populations confined to local environments where pollution is persistent and selection is blind to nonpolluted environments, selection may favour alleles that produce costs by enhancing survival in polluted environments at the cost of reducing performance in nonpolluted environments (Crow, 1957; Kliot & Ghanim, 2012; Morgan et al., 2007). Genes with such antagonistically pleiotropic effects are typically thought to underlie costs of adaptation to local environments (Falconer & McKay, 1996; Kawecki et al. 1997; Williams, 1957). However, in broadly distributed populations that undergo selection in both polluted and nonpolluted patches, selection should remove antagonistically pleiotropic alleles (Kassen, 2002; Sexton et al., 2017), and fitness costs of pollution tolerance should be eroded (Bono et al., 2017; Futuyma & Moreno, 1988; Leroi et al., 1994; Kassen & Bell, 1998; Kassen, 2002). Therefore, while costs may often be observed in laboratory selection experiments where populations are selected under conditions of persistent pollution exposure, such costs may be absent in wild populations that encounter pollutants in much patchier ways (Morgan et al., 2007).

To understand the nature of fitness costs of pollution tolerance in populations experiencing variable or patchy exposures to pollutants, we need more case studies testing for tolerance costs in natural populations selected under highly heterogeneous environments, especially populations that are wide ranging and highly dispersive. From an applied evolution perspective, our understanding of fitness costs of pollution tolerance in populations that experience variable or patchy environments is relevant to management of widely distributed invasive species. Such species are typically highly dispersive, occupy diverse habitats and are known for their abilities to rapidly colonize disturbed anthropogenic environments (Croci et al., 2007; Goodwin et al., 1999; Jahner et al. 2011; Peacock & Worner, 2008). Many invasive insect species in particular are also known agricultural pests, and understanding the degree to which fitness costs constrain pollution tolerance in these populations is a central component of many pest management strategies developed to prevent the propagation of tolerance alleles (Denholm & Rowland, 1992; Lenormand & Raymond, 1998). Although fitness costs of tolerance have often been observed in insect pest populations selected under simplified laboratory environments (Abbas et al., 2012; Cao & Han, 2006; Carrière et al., 2009; Kliot & Ghanim, 2012; Shah et al., 2015; Shirley & Sibly, 2006; Wenes et al., 2006), fewer studies have tested for costs present in genotypes sampled directly from the wild. Therefore, the focus of the present study was to test for fitness costs of tolerance to a range of polluted environments in a wild‐caught population of the cabbage white butterfly (*Pieris rapae*). The cabbage white is a widely distributed and highly dispersive invasive insect that is found all over the world and is recognized as an agricultural pest whose larvae feed on host plants in the Brassicaceae family (Hill, 1987). Its global spread is among the best documented of any invasive insect and was first introduced to North America from Europe in the mid‐19^th^ century (Shen et al., 2016; Ryan et al., 2019; Scudder, 1887). This species frequently inhabits host plants growing in both disturbed human‐impacted areas as well as more pristine habitats, such as open valleys, mountaintops and forested areas (Klots, 1978). Due to its broad range and highly dispersive nature, the cabbage white experiences patchy or variable exposures to heavy metal pollution through larval consumption of host plants growing in agricultural fields affected by metal‐containing pesticides (Wang et al., 2009; Wolz et al., 2003) and in urban roadsides and weedy lots that experience metal pollution from residual lead paint or deposition from vehicle wear‐and‐tear (Lagerwerff & Specht, 1970; Wei & Yang, 2010).

In this study, we evaluated fitness costs of tolerance to a range of dietary zinc exposures in wild‐caught families of cabbage white butterfly caterpillars captured in Minnesota, USA. Zinc is a widespread heavy metal pollutant that can accumulate in soils near roadsides, abandoned lots and agricultural fields. Along with other heavy metals, zinc is a component of some pesticides, industrial combustion and residues of vehicle traffic **(**Jaradat & Momani, 1999; Karim et al., 2014; Lagerwerff & Specht, 1970; Montagne et al., 2007; Ramakrishnaiah & Somashekar, 2002; Wei & Yang, 2010). Metal pollution can transfer from soil to plant leaf tissue where it can be consumed by caterpillars foraging in disturbed urban or agricultural areas (Hladun et al., 2013, 2016; Verbruggen et al., 2009; Xun et al., 2017). Although zinc has important regulatory roles in enzyme production and immunity (Rink & Haase, 2007; Scrutton et al., 1971), it is toxic at higher levels, oxidizing DNA and interfering with enzymatic processes (Koch & Hill, 2017; McRae et al., 2016). We used an artificial diet approach in the laboratory to precisely quantify cabbage white caterpillar tolerance to elevated dietary zinc concentrations ranging from 168 to 1769 ppm. These concentrations fall within the range of zinc levels found in leaves of plants in the family Brassicaceae growing in metal‐contaminated soils (e.g. 100–2000 ppm leaf zinc concentrations of plants growing in soils ranging from 200 to 400 ppm zinc; Herrero et al., 2003; Purakayastha et al., 2008).

We tested whether cabbage white butterfly families with relatively greater tolerance to zinc pollution (measured as family‐level survival on a zinc‐polluted diet) faced costs in terms of reduced investment in components of fitness under nonpolluted conditions. Because fitness consists of multiple components, we tested for costs in a broad range of life‐history traits. Our key life‐history traits of interest for measuring fitness costs of zinc tolerance were egg number (i.e. total number of mature eggs in the ovary) and reproductive effort (egg number × egg size) of female butterflies at day two of adulthood. These metrics are likely closely linked to fitness in the cabbage white butterfly, as peak oviposition in this species occurs at 2–3 days into adulthood, and adults typically live less than 14 days (Suzuki, 1978). Thus, the production of fewer eggs by day two of adulthood would likely result in lower lifetime reproductive output. We also tested for fitness costs of zinc tolerance in terms of larval development time, growth rate, adult body size and egg size. Additionally, we tested for a potential trade‐off between zinc tolerance and investment in melanin, a molecule with diverse functional roles in insects, including immunity, ornamentation and thermoregulation (Stoehr, 2006).

## 2 METHODS

### 2.1 Origin of cabbage white butterfly families and egg collection

We captured wild, gravid female *Pieris rapae* in and around agricultural fields and community gardens on the University of Minnesota Saint Paul campus in July–August 2018. Thirty females were captured over a 6‐week period and were housed individually in cages (61 × 61 × 61 cm, Bug Dorm) in a greenhouse. Fifteen of the original 30 captured females died in the greenhouse before laying any eggs, so our experiment focused on offspring of 15 different females. The bottom surface of each cage was covered with a damp towel to maintain humidity. All females were given *ad libitum* access to 10% honey water provided in sponges, which were changed daily. Each female was provided with 3‐week‐old green cabbage plants (*Brassica oleracea* var. Earliana) in 10 × 10 × 10 cm pots for oviposition. We removed each plant daily and counted the total number of eggs laid by each female. Upon removal, plants were transferred from their pots to 946‐ml plastic cups and housed in a climate chamber maintained at 25°C on a 14‐h photoperiod until hatched larvae were transferred to artificial diet.

### 2.2 Larval rearing on zinc‐polluted diet

All larvae were reared on an artificial diet (Troetschler et al., 1985) containing the following ingredients: 15 g cabbage flour, 50 g wheat germ, 10 g cellulose, 27 g casein, 24 g sucrose, 9 g Wesson's Salt Mixture, 12 g Torula yeast, 3.6 g cholesterol, 10.5 g Vanderzant Vitamin mix, 0.75 g methyl paraben, 1.5 g sorbic acid, 3 g ascorbic acid, 0.175 g streptomycin and 5 ml flaxseed oil. 15 g of fine mesh agar was boiled in 400 ml distilled water, then cooled by adding an additional 400 ml distilled water. All ingredients were then mixed in an industrial food blender.

Cabbage white larvae were reared on one of four artificial diet treatments prepared by adding either 0, 142.5, 285 or 2280 µl of 1 M ZnCl_2_ solution to the diet mix before blending. Dietary zinc concentrations were measured using the ICP‐AES technique at the University of Minnesota Analytical Research Lab (as average of two samples for each diet type). The final average zinc concentrations with standard deviations for each treatment were the following: 62 ± 0.51 ppm (control), 168 ± 0.71 ppm, 275 ± 0.85 ppm and 1769 ± 0.33 ppm. Zinc concentration of the control (62 ppm) treatment is within the range of Brassicaceae collected from nonpolluted field sites (Watanabe et al., 2007). Additionally, our zinc pollution treatments fall within the range of zinc concentrations found in leaves of Brassicaceae plants growing in metal‐polluted soils. For instance, zinc concentrations in metal‐polluted soils have been shown to range from 100 to 3000 ppm (Bryan & Hummerstone, 1973; Donker et al., 1996), and Brassicaceae plants grown in soils with 200–400 ppm zinc pollution have leaf zinc concentrations of 100–2000 ppm (Herrero et al., 2003; Purakayastha et al., 2008).

Seven days after egg collection, larvae were assigned to one of the four dietary treatments (20 larvae per family per treatment). Artificial diet was provided in 118‐ml plastic cups (2–3 larvae transferred per cup). All diet cups were stored in a walk‐in climate chamber maintained at 24°C with a 14‐h photoperiod. Larvae were left inside cups for the duration of the larval and pupal stage. Ten of the 15 captured females produced enough offspring to meet the sample sizes required for all four of our experimental treatments. However, the total number of families sampled in each individual treatment was the following: 15 (62 ppm), 12 (168 ppm), 11 (275 ppm) and 14 (1769 ppm).

### 2.3 Measurements of phenotypic performance

Survival, adult body size, development time and growth rate were measured in individuals from all four zinc treatment groups (62, 168, 275 and 1769 ppm). Survival was defined as whether an individual successfully transitioned from the larval stage to adult eclosion with wings fully intact (*n* = 973 individuals assess for survival in total). Development time was assessed as the time from egg collection to adult eclosion (*n* = 797 individuals were assessed given survival rates). Upon eclosion, all individuals were numbered on the hindwing with a black marker (assigned numbers were not correlated with zinc treatment, so adult handlers were blind to treatment). Females were transferred to a greenhouse for 48 h to allow for egg development before freezing at −20°C in airtight containers for subsequent dissections. Adult body size was measured by removing and photographing one forewing from each individual. Forewing length, measured using ImageJ (NIH), was quantified as the distance between the forewing apex and the articulation of the wing with the thorax (*n* = 540 individuals). Similar to other studies (Shephard et al., 2020; Sikkink et al., 2020), we measured the growth rate of each individual as forewing length divided by development time (*n* = 540 individuals).

Egg number and size were measured in females from the control (62 ppm) treatment only. Egg number was measured as the total number of mature eggs in the ovaries of females allowed to develop eggs for a period of 2 days after eclosion (*n* = 49 individuals). To measure egg size, we quantified the average length of 3–5 eggs from each female using ImageJ (*n* = 47 individuals). All ovary dissections were performed in 1× PBS buffer under a Leica M165C microscope at 10× magnification.

We measured wing melanin investment in adult female butterflies, given that females have larger and darker forewing spots compared to males. Wing melanin investment was measured in females from the control (62 ppm) and 1769 ppm zinc treatments only, since these were the best sampled treatments at the family level. All females used for wing melanin measurements were sacrificed 48 h after adult eclosion to control for any age‐specific effects of wing wear and tear. To measure wing melanin investment, a single forewing was removed from each female and imaged dorsally under controlled light conditions with a grey colour standard. A Canon Rebel T3 camera with a 50‐mm macro lens was used to capture all wing images. Wing melanin investment was measured in two ways: proportion of total wing melanization (*n* = 181 individuals) and wing melanin darkness (*n* = 120). To measure total proportion of wing melanization, we calculated the ratio of black to white coloration on one forewing using ‘pavo 2.0’ R package. ‘pavo 2.0’ allowed us to classify image pixels into predefined colour classes (black or white in our case) and calculate the relative proportion of the image covered by pixels of a given colour class. We used the proportion of black as a measure of proportion of wing melanization. To measure wing melanin darkness, we used an image‐processing algorithm in MATLAB that quantified average red (R), green (G), blue (B) and grey (Gy) values from the upper dorsal black spot of each wing. We controlled for possible differences in light conditions by standardizing and equalizing images using reflectance values and average red (R), green (G), blue (B) and grey (Gy) values of four grey, one black and one white colour standard on X‐Rite Color Checker Classic Mini. With this method, Gy values can range from 0 (black) to 255 (white), meaning that lower values correspond to darker, more melanized wing spots.

### 2.4 Statistical analysis

RStudio version 3.5.1 (RStudio Team, 2018) was used for all statistical analyses. We tested for effects of zinc treatment, family and the interaction between zinc treatment and family on survival using a generalized linear model with a binomial distribution and logit link function. Given that survival was used as the metric of zinc tolerance (see below), we were specifically interested in family‐level variation for survival across zinc treatments. Family was therefore treated as a fixed effect for the survival analysis. No random effects were included in this model.

We used a linear modelling approach to test for the effect of zinc treatment on development time, growth rate and adult forewing length (Table 1). In each model, ‘treatment, ‘family’ and ‘sex’ were included as fixed effects. We did not test for any interactive effects between treatment and sex because we did not have a priori expectations for these interactions. ‘Family’ was included as a fixed effect rather than a random effect in these models because we did not sample individuals from all 15 families across the four zinc treatments (see above). We also tested for the effect of zinc treatment on both wing melanin darkness and proportion of total wing melanization. In these models, ‘family’ was included as a random effect because wing melanization was measured in only the control and 1769 zinc treatments (see above).

**TABLE 1:** Summary of linear models testing for effects of dietary zinc treatment on phenotypic traits in the cabbage white butterfly (*Pieris rapae*)

	*N*	Zinc treatment	Family	Sex
Development time	797	*F*_3,791_ = 32.75, *p* < 0.001	*F*_1,791_ = 21.59, *p* < 0.001	*F*_1,791_ = 0.051, *p* = 0.82
Forewing length	540	*F*_3,534_ = 15.95, *p* < 0.001	*F*_1,534_ = 41.09, *p* < 0.001	*F*_1,534_ = 76.87, *p* < 0.001
Growth rate	540	*F*_3,534_ = 25.62, *p* < 0.001	*F*_1,534_ = 0.53, *p* = 0.47	*F*_1,534_ = 3.96, *p* = 0.047

Note

Each model tests for effects of dietary zinc treatment on a phenotypic trait, controlling for effects of family and sex.

To test for fitness costs of tolerance, we quantified relationships between family‐level zinc tolerance across dietary zinc treatments and phenotypic performance. Here, zinc tolerance was quantified as the percentage of individuals surviving from larva to adult eclosion with wings intact from each family in a given zinc treatment group. Analyses of trade‐offs can be confounded by differences in organismal condition (van Noordwijk & de Jong, 1986), which may stem from a range of factors, including differences in genetic quality, transgenerational effects or the ability to assimilate dietary resources. In our study, a likely source of variation in condition is how well cabbage white families perform on the control diet. To account for this, we calculated *relative survival* as the difference between survival on each elevated zinc treatment (i.e. 168, 275 or 1769 ppm) and survival on the control treatment (62 ppm) for each family. This controls for any family‐level differences in survival on the control diet that could bias inferences of tolerance on the elevated zinc treatments. We tested for fitness costs of tolerance by measuring average family‐level investment in six life‐history traits and two traits related to melanin investment under control conditions. Life‐history traits considered were egg number (the average number of mature eggs in ovaries of females from each family; 4–6 individuals per family, average =4.9), egg size (the average length of eggs sampled from females in each family; 4–5 individuals per family, average = 4.7), reproductive effort (the product of egg number and egg size for each female), development time (the number of days from egg collection to adult emergence; 14–20 individuals per family, average = 18.8), adult body size (estimated by adult forewing length; 11–18 individuals per family, average = 12.4) and growth rate (body size divided by development time; 10–18 individuals per family, average =12.3). Melanin investment traits considered were proportion of total wing melanization and wing melanin darkness (4–14 individuals per family, average = 7.7). Analyses of egg number, egg size, reproductive effort and proportion of total wing melanized were performed using residuals from a regression between forewing length (i.e. body size) and each of these variables across all individuals.

In our main tests for fitness costs of tolerance (Table 2), we used linear modelling approaches to test whether families with greater zinc tolerance would face costs under control conditions. In each analysis, the predictor variable was relative survival (i.e. zinc tolerance). Given that we were interested in testing for general costs of zinc tolerance rather than costs of tolerance to each specific level of zinc pollution, we present zinc tolerance values as family‐level least square means calculated from a linear model that included relative survival as a response variable and treatment and family as predictor variables. The response variable for each fitness cost analysis was a single phenotypic trait (either one of the six life‐history traits or two melanin investment traits listed above).

**TABLE 2:** Summary of linear models testing for relationships between relative zinc tolerance and investment in phenotypic traits across families of the cabbage white butterfly (*Pieris rapae*)

	Estimate	*SE*	*t*	*p*	*R* ^2^
Effect of relative zinc tolerance on average family reproductive effort (relative to body size)					
(Intercept)	1.15	2.26	0.66	0.52	0.39
Relative zinc tolerance	32.14	11.07	2.90	0.012	
Effect of relative zinc tolerance on average family egg number (relative to body size)					
(Intercept)	1.72	2.41	0.71	0.49	0.50
Relative zinc tolerance	42.90	11.81	3.63	0.003	
Effect of relative zinc tolerance on average family egg size (relative to body size)					
(Intercept)	−4.60 × 10^−3^	7.90 × 10^−3^	−0.58	0.57	0.52
Relative zinc tolerance	−0.14	3.88 × 10^−2^	−3.72	0.003	
Effect of relative zinc tolerance on average family development time					
(Intercept)	25.85	0.35	74.39	<0.001	0.04
Relative zinc tolerance	−1.31	1.70	−0.77	0.45	
Effect of relative zinc tolerance on average family forewing length					
(Intercept)	2.24	1.32 × 10^−2^	169.85	<0.001	0.02
Relative zinc tolerance	−2.98 × 10^−2^	6.49 × 10^−2^	−0.46	0.65	
Effect of relative zinc tolerance on average family growth rate					
(Intercept)	8.80 × 10^−2^	1.43 × 10^−3^	61.74	<0.001	0.04
Relative zinc tolerance	5.36 × 10^−3^	7.00 × 10^−3^	0.766	0.46	
Effect of relative zinc tolerance on average family proportion of total wing melanisation					
(Intercept)	4.80 × 10^−3^	5.04 × 10^−3^	0.95	0.36	<0.01
Relative zinc tolerance	−7.34 × 10^−4^	2.47 × 10^−2^	−3.00 x10^−2^	0.98	
Effect of relative zinc tolerance on average family wing melanin darkness					
(Intercept)	2.20 × 10^−2^	1.33 × 10^−3^	16.54	<0.001	0.01
Relative zinc tolerance	2.68 × 10^−3^	6.53 × 10^−4^	0.411	0.69	

Note

Each model tests for the effect of family‐level least square mean values for zinc tolerance (i.e. proportion surviving from larva to adult eclosion in zinc treatment relative to the control treatment) on family‐level investment in a phenotypic trait (i.e. a life‐history trait or melanin investment trait).

## 3 RESULTS

### 3.1 Family‐level variation for heavy metal tolerance

**FIGURE 1:**
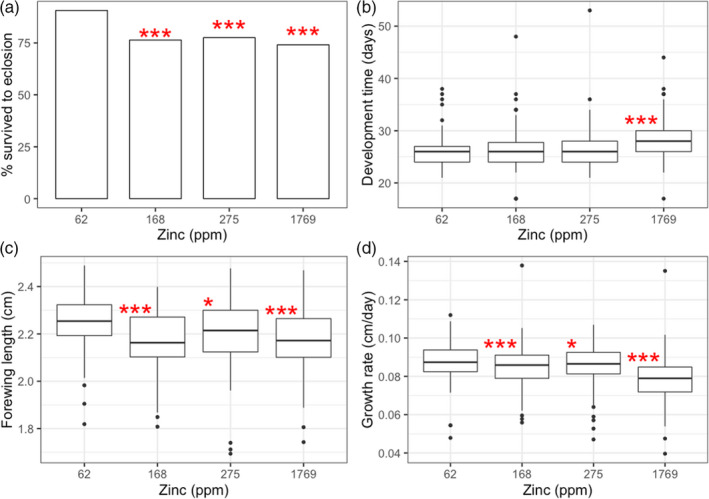
Effects of dietary zinc exposure in cabbage white butterfly larvae (*Pieris rapae*) on (a) survival from larva to adulthood, (b) development time to adulthood (days), (c) adult forewing length (cm) and growth rate (cm/day). We quantified survival as the percentage of larvae in each dietary zinc treatment reaching eclosion (i.e. adulthood) with wings fully intact. Development time was quantified as the number of days from the date eggs were laid until eclosion. Forewing length (an estimate of adult body size) was measured as the distance between the apex of the forewing and the articulation of the forewing with the thorax. Growth rate was measured as forewing length divided by development time. Asterisks indicate a significant difference relative to the control (no zinc added; 62 ppm) treatment (*p < 0.05, **p < 0.01, ***p < 0.001). Note that the survival data in (a) show percentage of individuals survived for visualization purposes and does not reflect the statistical tests described in the Methods. Boxplot details: middle line shows median, box lengths shows interquartile range, whiskers show lower and upper quartiles, and points show outliers

**FIGURE 2:**
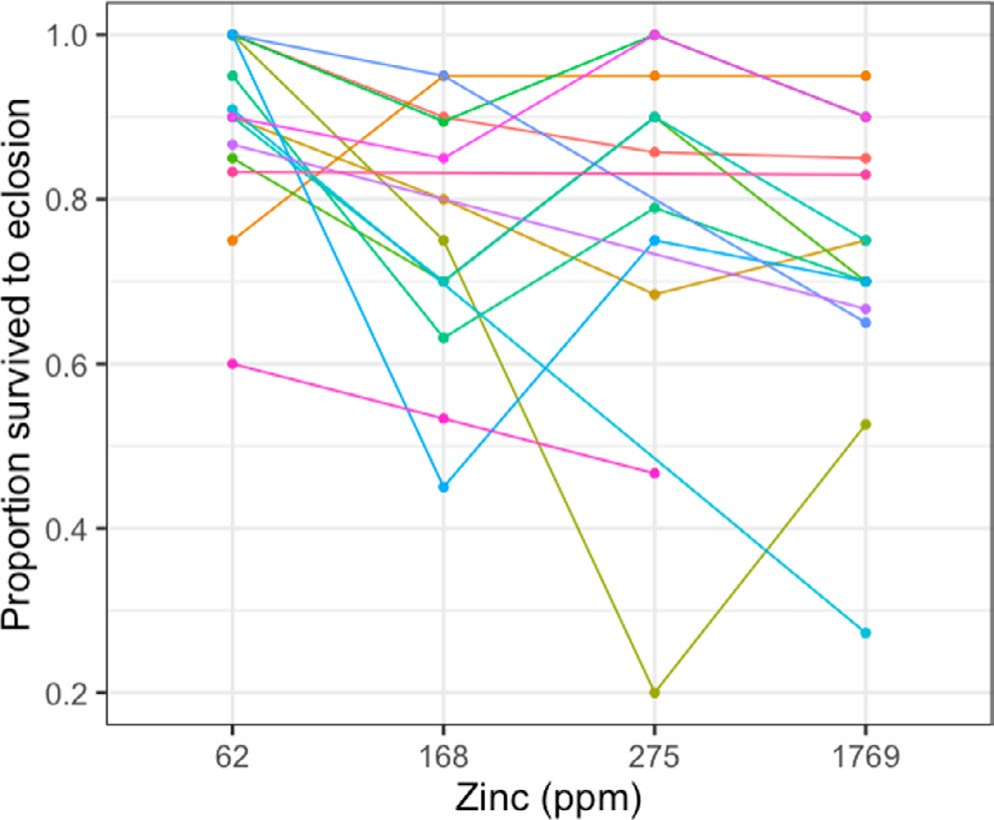
Family‐level reaction norms for tolerance across concentrations of dietary zinc exposure in larvae of the cabbage white butterfly (*Pieris rapae*). Zinc tolerance was quantified as the proportion of larvae in each dietary zinc treatment surviving to eclosion (i.e. adulthood) with wings fully intact across four dietary zinc treatments: 62 ppm (control; no zinc added to diet), 168, 275 and 1769 ppm. Each reaction norm represents a different cabbage white butterfly family

Heavy metal tolerance was quantified as the proportion of individuals per family surviving from larva to adult eclosion with wings fully intact in each dietary zinc treatment (Figures 1a and 2). Survival differed across dietary zinc treatments (treatment: *X* ^2^ = 26.74, *p* < 0.001; family: *X* ^2^ = 4.50, *p* = 0.03), but the treatment‐by‐family interaction for survival was not significant survival (*X* ^2^ = 5.40, *p* = 0.14). Relative to the control (62 ppm) treatment, survival was ~14% lower in the 168 ppm treatment (*X* ^2^ = 19.61, *p* < 0.001), ~13% lower in the 275 ppm treatment (*X* ^2^ = 16.11, *p* < 0.001) and ~16% lower in the 1769 ppm treatment (Figure 1a; *X* ^2^ = 24.85, *p* < 0.001).

### 3.2 Effects of heavy metal exposure on components of fitness

We quantified effects of elevated zinc exposure on investment in life‐history traits. There were significant effects of zinc treatment on cabbage white development time, forewing length and growth rate (Table 1). Relative to the control treatment (62 ppm), development time was longer in the 1769 ppm treatment by ~2.5 d (Figure 1b; *t* = 8.25, *p* < 0.001). Compared to the control (62 ppm) treatment, forewing length was shorter in the 168 ppm, 275 ppm and 1769 ppm treatments by ~0.07 cm (Figure 1c; *t* = −4.79, *p* < 0.001), ~0.04 cm (*t* = −2.73, *p* = 0.01) and ~0.07 cm (*t* = −5.04, *p* < 0.001), respectively. Relative to the control treatment, growth rate was slower in the 168 ppm, 275 ppm and 1769 ppm treatments by ~0.003 cm/day (Figure 1d; *t* = −2.75, *p* = 0.006), ~0.002 cm/day (*t* = −1.95, *p* = 0.05) and ~0.01 cm/day (*t* = −8.30, *p* < 0.001), respectively.

### 3.3 Fitness costs of heavy metal tolerance

**FIGURE 3:**
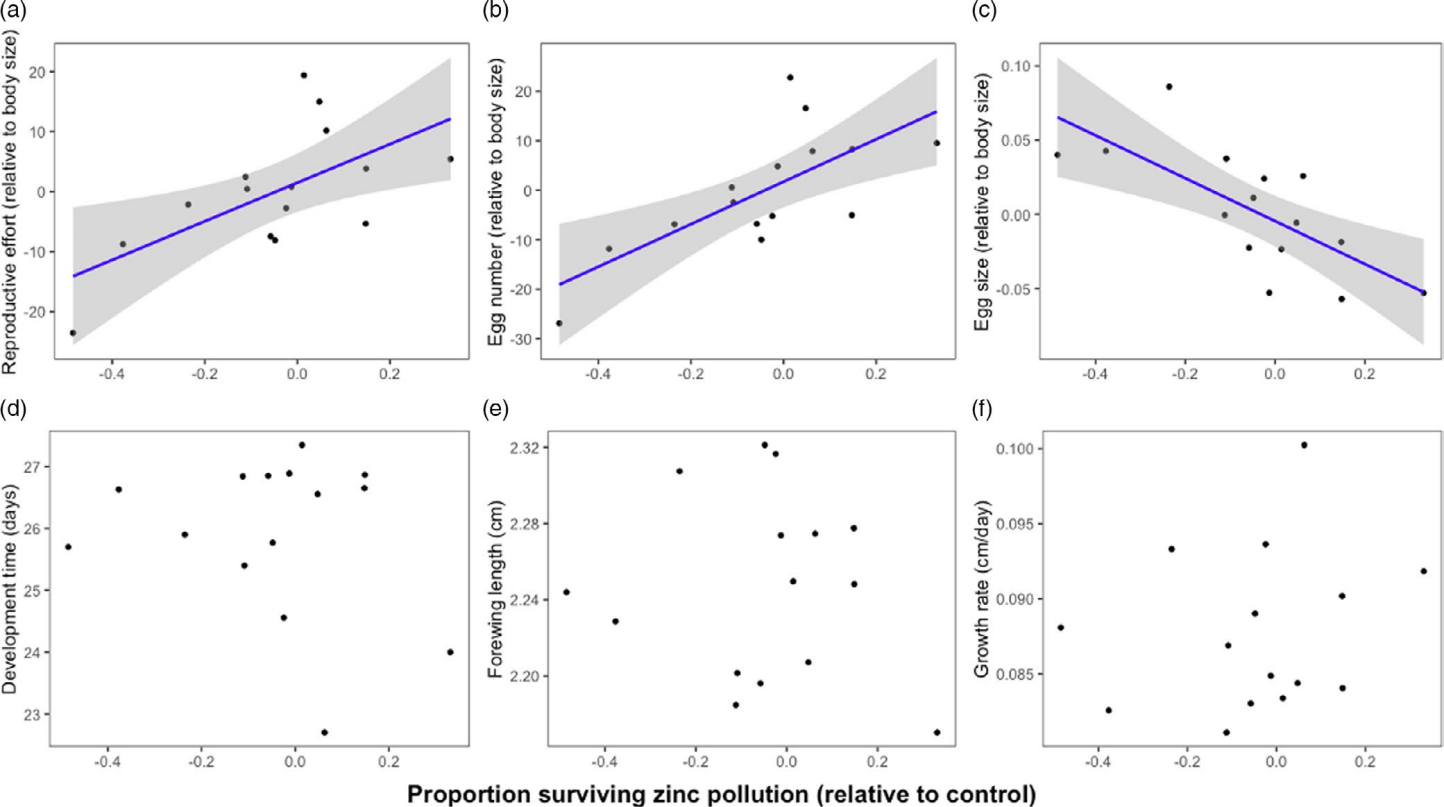
Relationships between relative zinc tolerance (i.e. survival to adulthood) and average family reproductive effort (a), egg number (b), egg size (c), development time (d), forewing length (e) and growth rate (f) across families of cabbage white butterflies (*Pieris rapae*). Each point corresponds to offspring from a single cabbage white butterfly family. Zinc tolerance is presented as family least square mean values from a linear model testing for effects of zinc treatment and family on relative survival across families. Here, relative survival is quantified as proportion of larvae survived from larva to adult eclosion in each elevated zinc treatment (168, 275 or 1769 ppm) relative to the control treatment (no zinc added; 62 ppm). We show relative survival values here because they account for differences in family survival on the control diet, which could confound effects of zinc tolerance (see Methods). Reproductive effort (a) was calculated as the product of egg number and egg size for each female. Egg number (b) was estimated by counting the total number of mature eggs in the ovaries of dissected females 48 h after eclosion. Egg size (c) was estimated by measuring the average length of mature eggs dissected from female ovaries 48 h after eclosion. Development time (d) was quantified as the number of days from the date eggs was laid until eclosion. Forewing length (e; an estimate of adult body size) was measured as the distance between the apex of the forewing and the articulation of the forewing with the thorax. Growth rate (f) was measured as forewing length divided by development time. Family averages for egg size and number are calculated using residuals from regression analyses between forewing length (our metric for adult body size) and either egg size or number among all individuals from all families

To test for fitness costs of zinc tolerance, we evaluated whether higher relative survival across dietary zinc treatments at the family level was associated with lower investment in life‐history traits under control conditions. We found that families with higher relative survival on zinc pollution produced more eggs and had higher reproductive effort (Figure 3a,b). Yet, families with higher relative survival on zinc pollution produced smaller eggs (Figure 3c). Indeed, there was a negative relationship across families between egg number and egg size under control conditions (*F*_1,13_ = 5.50, *p* = 0.03, *R* ^2^ = 0.30; Figure S1). There were no significant relationships between relative survival on zinc pollution and life‐history trait investment for either development time, forewing length or growth rate (Table 2, Figure 3d–f).

### 3.4 Effects of heavy metal exposure on wing melanization:

**FIGURE 4:**
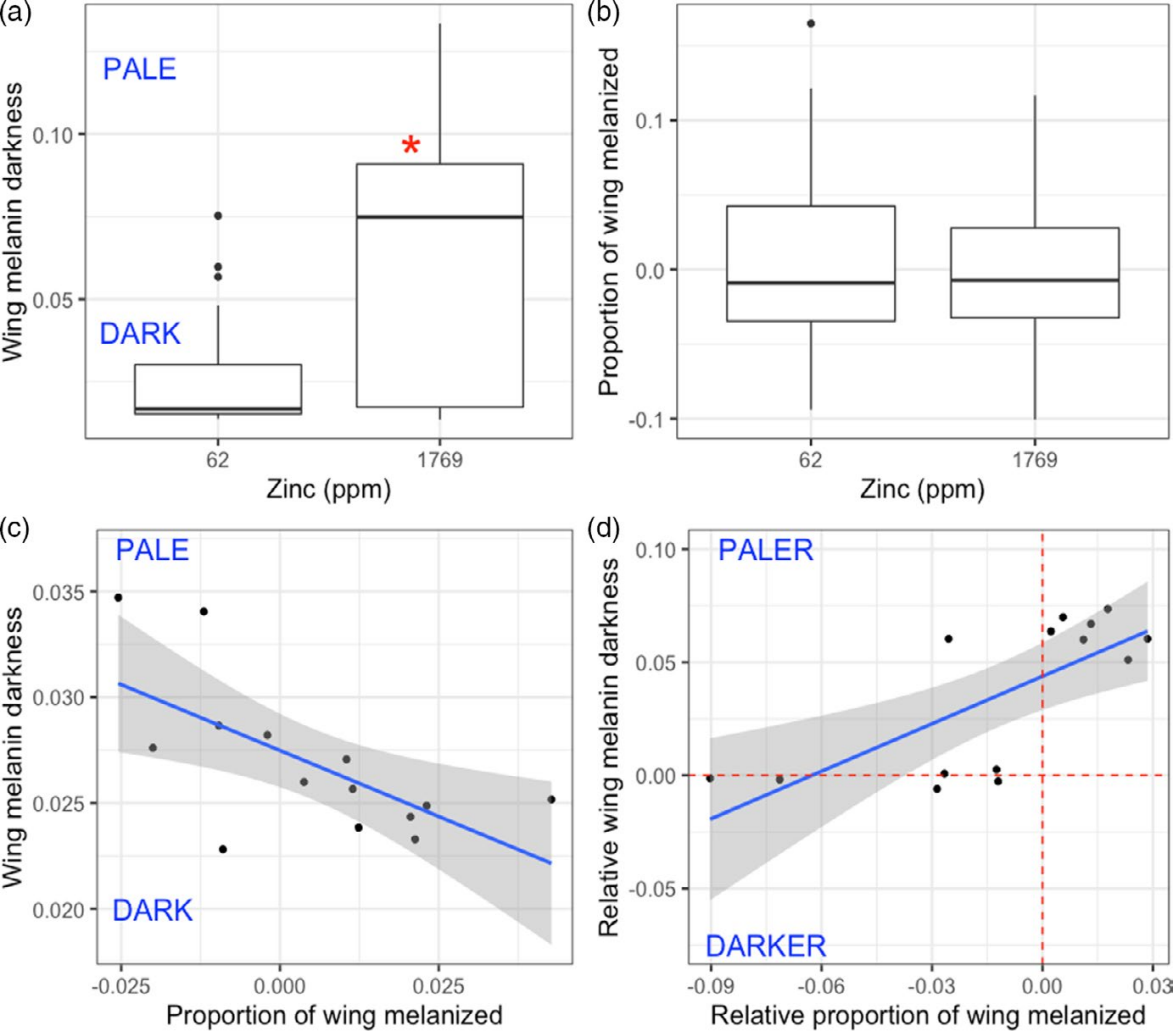
Effects of larval zinc exposure on adult wing melanization in the cabbage white butterfly (*Pieris rapae*). Larvae were exposed to one of two dietary zinc treatments: control (62 ppm, no zinc added to diet) or elevated (1769 ppm). Wing melanin investment was quantified in two ways: darkness of the upper melanic wing spot (a) and proportion of total wing melanization (b). Panel C shows the relationship between proportion of total wing melanization and melanin wing darkness across families under control conditions. Panel D shows the relationship between both measures of melanin investment under conditions of elevated zinc exposure relative to control conditions. For instance, a point in the top right quadrant indicates that a family developed a relatively higher proportion of total wing melanization but relatively paler wings in the elevated zinc condition compared to its development in the control condition. Boxplot details: middle line shows median, box lengths shows interquartile range, whiskers show lower and upper quartiles, and points show outliers. Proportion of wing melanized values in (b) are presented as residuals from a regression between forewing length and proportion of wing melanized among all individuals from all families. Family averages for proportion of wing melanized (c) and relative proportion of wing melanized (d) are also calculating using the residuals from this analysis

We also tested whether elevated zinc exposure constrained investment in wing melanin production. Given that wing melanin may vary with respect to total area of melanin coverage or darkness of melanized patches, we measured melanin investment in two ways: proportion of total wing melanized and melanin wing patch darkness. Relative to control conditions (62 ppm zinc), individuals exposed to elevated zinc conditions (1769 ppm) developed less melanized wing patches (i.e. paler black coloration), on average (*t* = 13.78, *p* < 0.001, Figure 4a). However, proportion of total wing melanization did not differ between control and elevated zinc conditions (*t* = −0.41, *p* = 0.7, Figure 4b). Under control conditions (62 ppm zinc), families with a greater proportion of total wing melanization also developed darker wing patches (Spearman's rho = −0.69, *p* = 0.008, Figure 4c). However, under conditions of elevated zinc exposure (1769 ppm), families that developed a relatively greater proportion of total wing melanization also developed paler wing patches (Spearman's rho = 0.69, *p* = 0.008, Figure 4d).

We also tested whether investment in wing melanization imposed a cost of zinc tolerance by focusing on two traits related to melanin investment: wing melanin darkness and proportion of total wing melanization. However, we found no significant relationships between relative survival on zinc pollution and either trait related to melanin investment (Table 2).

## 4 DISCUSSION

Genotypes that can better tolerate pollutants are expected to face fitness costs in nonpolluted environments, yet such costs are not always detected in empirical studies. One possible reason is that costs may be selected against in populations that experience highly variable exposures to pollutants across space or time. In such environments, characterized by mosaics of polluted and nonpolluted patches, selection may erode costs and favour genotypes that perform well in both types of environments. This situation may be relevant to wide ranging pest populations, which are known to colonize diverse polluted and nonpolluted environments. We therefore tested for fitness costs of tolerance to a range of heavy metal‐polluted environments in wild‐caught families of a widely distributed invasive pest species, the cabbage white butterfly (*Pieris rapae*). We found that cabbage white families that were more tolerant to zinc pollution had greater reproductive effort and higher fecundity under nonpolluted conditions (Table 2; Figure 3), suggesting that this widely distributed wild population does not have costs of tolerance commonly observed in populations confined to local environments where pollution exposure is persistent.

Understanding the degree to which fitness costs constrain pollution tolerance in populations of invasive pest species is crucial to the development of strategies to mitigate the spread of tolerant genotypes (Denholm & Rowland, 1992; Lenormand & Raymond, 1998). Although fitness costs of pollution tolerance have been observed in insect pest populations, most studies have focused on laboratory‐selected populations rather than genotypes sampled directly from the wild (Abbas et al., 2012; Cao & Han, 2006; Carrière et al., 2009; Kliot & Ghanim, 2012; Shah et al., 2015; Shirley & Sibly, 2006; Wenes et al., 2006). In laboratory selection regimes, populations typically encounter relatively constant pollutant levels over multiple generations where selection is blind to nonpolluted environments. In these conditions, fitness costs of tolerance likely emerge due to selection for antagonistically pleiotropic alleles that enhance fitness in polluted environments but negatively impact fitness in nonpolluted environments (Crow, 1957; Kliot & Ghanim, 2012; Morgan et al., 2007). However, under more natural conditions where populations likely encounter variable exposures to both polluted and nonpolluted patches across space and time, selection may eliminate pleiotropic mutations whose effects enhance fitness in one environment but reduce fitness in the other (Kassen, 2002; Sexton et al., 2017). As a highly dispersive, wide ranging, invasive pest species, our cabbage white butterfly study population experiences selection with highly variable or patchy exposures to heavy metal pollution across space or time by inhabiting a combination of metal‐polluted anthropogenic habitats (e.g. roadsides and agricultural fields) as well as more pristine habitats, such as open valleys and mountaintops (Shen et al., 2016). Such patchy selection should select against pollution specialist genotypes and favour generalists that thrive in both polluted and nonpolluted habitats (Futuyma & Moreno, 1988; Leroi et al., 1994; Kassen & Bell, 1998; Kassen, 2002; Bono et al., 2017). Although this work considers a population of only a single species, our results combine with previous case studies suggesting that in populations that are highly dispersive or widely distributed, between‐environment fitness costs may be weak or absent (Agra et al., 2011; Sikkink et al., 2020) or positive relationships between fitness components may emerge (Niitepõld & Hanski, 2013). Future meta‐analytic approaches may help provide a more comprehensive test of how costs of pollution tolerance are moderated by environmental heterogeneity or range size.

Although zinc tolerance was positively related to both reproductive effort and egg number (Figure 3a,b), we found that zinc tolerance was negatively related to egg size (Figure 3c). These patterns are consistent with the idea that heavy metal tolerance may hinge on the common life‐history trade‐off between offspring size and number (Stearns, 1992), where families with reduced investment per offspring (i.e. smaller eggs) have lower tolerance. Indeed, we observed a trade‐off between egg size and number across cabbage white families under control conditions (Figure S1). If smaller eggs are linked to reduced offspring quality, it is possible that more heavy metal‐tolerant families may have a reduced ability to compete for resources such as food or mates (Parker & Begon, 1986; Sibly & Calow, 1989), which could be costly under certain field conditions that are seldom replicated in laboratory experiments (Agrawal, 2001; Van Buskirk & Steiner, 2009). Reduced competitive ability of organisms that are better adapted to coping with stressful or disturbed environments is a trade‐off that is central to Grime's universal adaptive strategy theory (Grime, 1977). Grime hypothesized that ruderal strategists, namely those that are short‐lived colonizers with high reproductive output, should have greater success in harsh, frequently disturbed environments. However, under more benign environments, these strategists should be outcompeted by larger, more resource‐efficient variants. As a short‐lived insect pest species that is invasive to North America (Hill, 1987), the cabbage white butterfly exhibits characteristics that are consistent with Grime's ruderal strategist. The cabbage white frequently colonizes disturbed habitats such as agricultural crops and ruderal vegetation in empty lots or along roadsides (Shen et al., 2016). As such disturbed areas may commonly coincide with the presence of anthropogenic chemical contaminants (e.g. agricultural pesticides or heavy metals from traffic residue), the ecology of the cabbage white butterfly may preadapt it to successfully colonize polluted or stressful environments, perhaps at the cost of a reduced ability to compete for resources under more benign conditions. Although key variables such as competitive ability and dispersal ability were not measured in our study, future approaches incorporating these ecologically relevant parameters may provide novel insights into life‐history variation associated with adaptation to polluted environments, particularly in highly dispersive invasive species.

While exposure to zinc pollution negatively impacted overall cabbage white survival at all levels tested (Figure 1a), there were a few cases in which individual families responded positively to zinc pollution (Figure 2). Although these positive effects of zinc pollution on survival may be attributed to noise, it is also possible that they could be a result of hormesis—a phenomenon where low‐level stress exposures can have positive or stimulatory effects on organismal performance (Calabrese & Baldwin, 2003; Costantini et al., 2010). Hormetic effects are thought to be in part mediated by protective physiological stress responses, such as antioxidants and heat shock proteins, which become activated in response to mild stressors (Costantini et al., 2012; Sørensen et al., 2007). While hormesis has been observed in diverse taxa, it has been particularly well studied in insect pest species and has been implicated as a potential mechanism whereby such species may adapt to stressful or polluted environments (Cutler, 2013; Guedes & Cutler, 2014).

We found no evidence for a cost of zinc tolerance in terms of melanin production (Table 2), but we did find that zinc exposure negatively impacted cabbage white wing melanization. Cabbage white larvae exposed to elevated dietary zinc developed paler black coloration on adult wing spots (Figure 4a), consistent with previous observations that larval stress reduces melanin production in adult butterflies (Talloen et al., 2004). Additionally, while we observed a positive correlation among families between proportion of total wing melanization and wing melanin darkness under control conditions (Figure 4c), we found the opposite relationship under conditions of elevated zinc exposure (Figure 4d): families developing a relatively higher proportion of total wing melanization under elevated zinc conditions only did so at the expense of developing relatively paler wings. As melanin plays diverse roles in insect immunity, thermoregulation and sexual signalling (Stoehr, 2006), it is possible that reduced melanin production under heavy metal stress could negatively impact these functions. However, it should be noted that this reduction of melanin production under heavy metal stress is inconsistent with previous observations of increased melanin pigmentation in metal‐tolerant populations of birds (Giraudeau et al., 2015) and reptiles (Goiran et al., 2017). Melanization is a hypothesized physiological mechanism of heavy metal tolerance, given that melanin readily binds heavy metals and may reduce their accumulation in biological tissue by storing them in dead pigmented cells (e.g. feathers, skin or scales) where they can be excreted (McGraw, 2003). It is possible that melanin may play less of a role in insect heavy metal tolerance relative to vertebrates, where most evidence for a link between melanization and heavy metal detoxification has been obtained. For instance, insects and vertebrates differ in several key components of the melanin synthesis pathway (Stoehr, 2006), and insects use melanin for a variety of functions that vertebrates do not, such as immunity (Barnes & Siva‐Jothy, 2000; True, 2003) and cuticle hardening (Sugumaran, 1998). Such factors may limit the ability for melanin to aid in heavy metal tolerance in insects.

This study has some limitations that could be addressed in future work. For instance, while it is likely that much of the variation in heavy metal tolerance observed in our study is due to standing genetic variation in the local population, we were unable to account for other factors that may contribute to this variation, such as maternal effects (Mousseau & Fox, 1998). Environmentally induced maternal effects have been shown to have a large influence on cadmium tolerance in the snail, *Brotia hainanensis* (Lam, 1996). Even though all families used in our study originated from gravid females captured in the same local area during the same 6‐week period, we cannot rule out the possibility that larval zinc tolerance could have been partially influenced by differences in maternal age, experience, or other environmentally induced factors that could affect offspring performance. Additionally, we cannot rule out the possibility that the positive relationship we found between egg number and zinc tolerance reflects a bet‐hedging strategy (Simons, 2011), where investing in a higher number of offspring may increase the probability of some of those offspring surviving in unpredictably variable environments. Moreover, our tests for fitness costs of zinc tolerance focused on only 15 butterfly families, a sample size that could be considered low for family‐level analyses. Large family sample sizes are extremely difficult to obtain in butterfly populations sampled directly from the wild, given limitations associated with rearing labour, number of females that can be captured during a field season and number of eggs that can be harvested from each female. Despite this limitation, we did observe significant relationships between zinc tolerance and fitness‐related traits (Figure 3) and have no evidence that statistical power is biasing the conclusions. Finally, it should be noted that in our study, all costs were measured in terms of relative fitness as opposed to absolute fitness. While absolute fitness can be extremely difficult to quantify in genotypes of wild‐caught populations, relative fitness measures are common in evolutionary biology (Bell, 2017). However, our measures of egg number and reproductive effort at two days into adulthood are likely closely linked to fitness in the cabbage white butterfly. Given that peak reproductive output in this species occurs at 2–3 days into adulthood, and typical adult lifespan in the field is less than 14 days (Suzuki, 1978), it is likely that producing fewer eggs by day two of adulthood would reduce lifetime reproductive output. Still, future studies testing for fitness costs in wild populations might consider using absolute fitness measurements (e.g. population growth rate) or aster modelling approaches to quantify lifetime fitness (Shaw et al., 2008).

In summary, we found no direct support for fitness costs of heavy metal tolerance in a wild cabbage white butterfly population. Rather, families that were more tolerant to heavy metal pollution had greater investment in egg number and reproductive effort under nonpolluted conditions (Figure 3a,b). Our case study raises a more general hypothesis of why fitness costs of pollution tolerance are not always observed in populations: patchy selection with pollutants should erode costs and favour genotypes that perform well under both polluted and nonpolluted conditions. We suggest that this might partly explain why widely distributed species are predisposed to become invasive in novel regions or habitats (e.g. agricultural habitats). Yet, the trade‐off we observed between heavy metal tolerance and egg size (Figure 3c) raises the possibility that more tolerant families may face costs in harsher field‐representative conditions if egg size is linked to offspring quality or competitive ability. Therefore, our results leave open the possibility that laboratory studies may sometimes fail to identify more subtle fitness costs in wild‐caught populations because such studies rarely replicate key ecological conditions encountered in the field (Van Buskirk & Steiner, 2009). As this may often be the case for laboratory‐based pest management research, we suggest that a productive way forward will be to test how costs of tolerance to polluted environments are influenced by ecologically relevant factors such as competition, food limitation, predation or parasitism (for instance, see Brady et al., 2017; Cothran et al., 2013; Hua et al., 2013; Jansen et al., 2011). Incorporating such ecological relevance into laboratory‐based toxicological research may provide new insights for managing the proliferation of pollution‐tolerant pest populations.

## CONFLICT OF INTEREST

The authors declare no conflict of interest.

## Supporting information

Figure S1

## Data Availability

Data will be archived on DRYAD.
