# Supplementary figures and images for: Evaluating costs of heavy metal tolerance in a widely distributed, invasive butterfly
Source: Evol Appl. 2021 Apr 3;14(5):1390–402. doi: 10.1111/eva.13208 (PMC8127708; doi:10.1111/eva.13208)

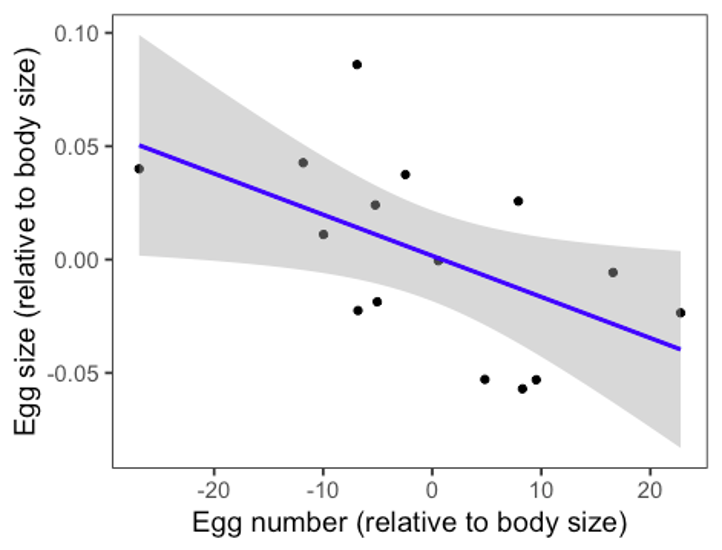

Supplement: Supplementary file 1 — Figure S1 [file EVA-14-1390-s001.png]
